# Design of anomalous reflecting metasurface for communication systems

**DOI:** 10.1038/s41598-024-82993-5

**Published:** 2025-01-03

**Authors:** Hany M. Zamel, Eman M. Eldesouki, Ahmed M. Attiya

**Affiliations:** 1 Microwave Engineering Department, Electronics Research Institute (ERI) Cairo, Cairo, Egypt; 2 School of Sciences and Engineering, American University in Cairo (AUC), Cairo, Egypt

**Keywords:** Metasurfaces, Genetic algorithms, Anomalously reflecting surface, Periodic structures, Engineering, Mathematics and computing

## Abstract

This paper presents a novel design approach for an anomalous reflector metasurface for communication systems operating at 8 GHz band. The main contribution of this work is the development of a general analytical method that accurately calculates the electromagnetic response of realistic metasurfaces with periodic impedance profiles. The modulated surface impedance is achieved by incorporating appropriately sized conductive patches on a grounded dielectric substrate. The proposed design utilizes a genetic algorithm (GA) optimization technique to optimize the surface impedance that achieving efficient reflection of incident waves towards a specific angle of 45˚. The optimization process targets a specific impedance profile derived from the analytical model, leading to the desired anomalous reflection behavior. Then by using periodic boundary conditions, dimensions of an elliptical unit cells can be obtained. To evaluate the anomalous reflection performance, the bistatic radar cross section (BRCS) are simulated at different frequencies. A reflector metasurface sheet is fabricated and measured for verification. The proposed approach provides a foundation for implementing intelligent metasurfaces in various communication applications.

## Introduction

The field of metasurfaces has experienced remarkable growth in recent years, offering unparalleled control over the behavior of electromagnetic waves at the nanoscale. Anomalously reflecting metasurfaces, in particular, have shown great potential in manipulating incident light waves for applications such as reflection, diffraction, beam steering, and cloaking^1–9^.

An anomalous reflector metasurface relies on a non-uniform, or modulated surface impedance. In other words, the surface impedance varies across its position, often following a periodic pattern. Anomalous reflectors typically achieve their function by tailoring the phase shift of the reflected wave at each location. This control is implemented through the use of specifically designed unit cell elements, like conductive patches, cavities, and lumped elements^10–13^.

A passive and lossless perfect reflector might be achievable by enabling controlled power transfer along the metasurface via surface waves. This approach would involve designing the modulation period so that different sections act as if they were lossy or equivalents. A phase-shifting approach, applied to design separately the necessary active and lossy parts of a nonlocal metasurface, is presented in^14^.

Yepes et al., introduced crucial Floquet modes, manifested as surface currents, which enable the cancellation of unwanted reflected waves in undesired directions. These Floquet modes also play a vital role in isolating the desired reflected wave. After extracting the local modulated reflection coefficient, the model is only applicable for achieving retroreflection^15^. The challenge was overcome by implementing a modulation with non-diagonal anisotropic elements.

Gradient metasurfaces can manipulate incident waves, either by reflecting them in unexpected directions or by controlling their propagation path, are presented in^16^. Unfortunately, the performance of gradient metasurfaces is sensitive to the polarization of the incident wave, restricting their applicability.

Barnard et al., presented a planar metasurface with phase gradients in both x and y directions will introduce two additional wave components to the reflected wave, causing it to propagate in a new direction^17^. These reflected waves from a phase gradient metasurface have a small magnitude and their direction can be calculated using the incident wave and phase gradient components. Malleboina et al. developed 1-bit and 2-bit digitally coded metasurfaces that use phase gradient digital unit cells to achieve anomalous reflection and steer normally incident plane waves in the desired direction^18^. A comprehensive study of four leading design techniques for anomalous reflectors is presented in^19^. This analysis is useful for practical applications, such as supercells composed of six metal patches on a grounded dielectric substrate, and the angular behavior of the designed metasurfaces is also investigated.

In this study, a simplified approach for designing modulated anomalous reflector metasurfaces is presented. The approach uses an analytical model to evaluate the electromagnetic behavior of metasurfaces with varying surface impedance. The GA was chosen to optimize the periodic impedance profiles of the modulated reflector. The modulated surface impedance is achieved by incorporating appropriately sized conductive patches on a grounded dielectric substrate. The design procedure can be applied for both transverse electric (TE) and transverse magnetic (TM) polarizations, allowing for compatibility with all possible incident plane waves. After optimizing the surface impedance, a method for designing the required reflecting surface with a prescribed surface impedance is presented. Simulations are conducted at frequency bands from 7.8 GHz to 8.2 GHz to assess the anomalous reflection performance, utilizing BRCS measurements using HFSS.

This paper is organized as follow. Second section outlines the necessary characteristics of a metasurface that functions as an ideal anomalous reflector. Then, introduces an analysis of a passive and lossless modulated periodic structure. To complete the analysis, the surface impedance is expressed as a Fourier series and the field components are formulated based on Floquet’s theorem. By applying the characteristic boundary condition to the metasurface, a solvable system of equations is obtained. Third section introduces the procedures for obtaining the surface impedance using GA. Fourth section discusses the analysis of the unit cell of the anomalous reflector. Fifth section presents the results of numerical simulations. Experimental verification and comparison with the simulated results are discussed in sixth section. Finally, the concluded remarks are the last section.

## Theoretical analysis

**Fig. 1:**
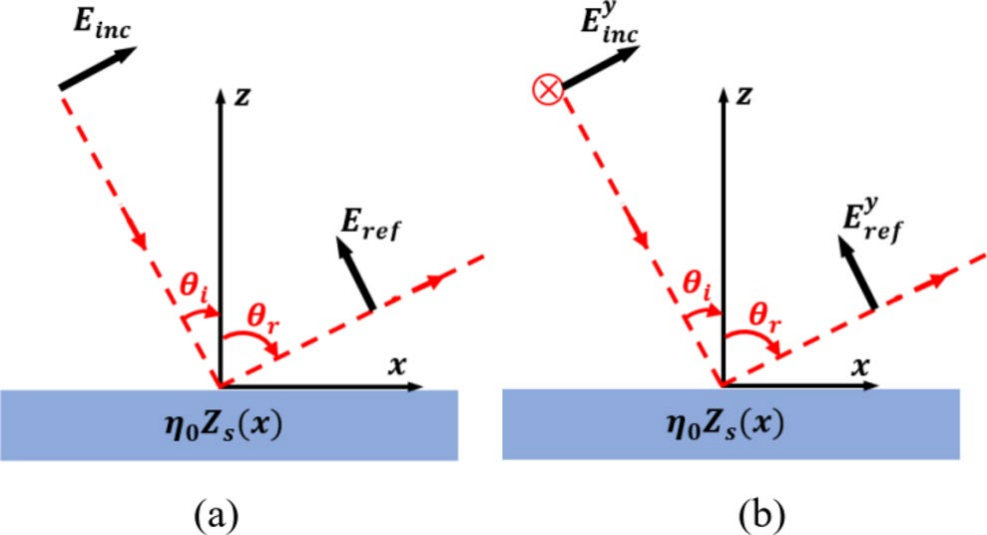
The incident and reflected field components for (**a**) TM and (**b**) TE polarization.

Consider infinitely planar thin metasurface lying on the $\:x-y$ plane. This metasurface has a surface impedance, $\:{\eta\:}_{0}{Z}_{s}\left(x\right)$, that varies periodically across its surface for TM and TE polarizations, as shown in Fig. 1. Here, $\:{\eta\:}_{0}$ is the characteristic impedance of free space, and $\:{Z}_{s}\left(x\right)$ denotes the normalized impedance of the metasurface, which also varies periodically along the $\:x-$direction. An incident plane wave ($\:{E}_{inc},\:{H}_{inc}$) interacts with a reflecting surface at an angle $\:{\theta\:}_{i}$ in the $\:xz$ plane. All incident power is reflected anomalously at an angle $\:{\theta\:}_{r}$ as another plane wave ($\:{E}_{ref},\:{H}_{ref}$), as shown in Fig. 1. The incident and reflected electric fields, denoted by $\:{E}_{\text{i}\text{n}\text{c}}$ and $\:{E}_{ref}$ respectively, are given by^20–22^:

1 $$\:{E}_{\text{i}\text{n}\text{c}}=\:{E}_{0}^{i}\:{e}^{-j{k}_{o}(\widehat{x}\text{sin}{\theta\:}_{i}+\widehat{y}\text{cos}{\theta\:}_{i})}$$

2 $$\:{E}_{\text{r}\text{e}\text{f}}=\:{E}_{0}^{r}\:{e}^{-j{k}_{o}(-\widehat{x}\text{sin}{\theta\:}_{r}+\widehat{y}\text{cos}{\theta\:}_{r})}$$

where,$\:\:{k}_{o}$ is the free space wave number and $\:{E}_{\text{r}\text{e}\text{f}}$ is given in term of $\:{E}_{\text{i}\text{n}\text{c}}$ as follows:

3 $$\:{E}_{\text{r}\text{e}\text{f}}=\:\sqrt{\frac{\text{cos}{\theta\:}_{i}}{\text{cos}{\theta\:}_{r}}}{E}_{\text{i}\text{n}\text{c}}$$

The boundary condition at the metasurface is expressed in terms of total tangential electric and magnetic field components, $\:{E}_{t}$ and $\:{H}_{t}$, and $\:{Z}_{s}$ as:

4 $$\:{\eta\:}_{0}{Z}_{s}\widehat{z}\times\:{H}_{t}=\:{E}_{t}$$

The dependence of surface impedance on the two cases of polarization TM and TE of the electromagnetic wave, is expressed through the following equations:

5 $$\:{Z}_{s}^{TM}=\:\frac{\sqrt{\text{cos}{\theta\:}_{i}\text{cos}{\theta\:}_{r}}\:\left(\sqrt{\text{cos}{\theta\:}_{i}}-\:\sqrt{\text{cos}{\theta\:}_{r}}\:{e}^{j{\Phi\:}\left(x\right)}\right)}{\sqrt{\text{cos}{\theta\:}_{r}}+\sqrt{\text{cos}{\theta\:}_{i}}\:{e}^{j{\Phi\:}\left(x\right)}}$$

6 $$\:\:\:{Z}_{s}^{TE}=\:\frac{\sqrt{\text{cos}{\theta\:}_{r}}+\:\sqrt{\text{cos}{\theta\:}_{i}}\:{e}^{j{\Phi\:}\left(x\right)}}{\sqrt{\text{cos}{\theta\:}_{i}\text{cos}{\theta\:}_{r}}\:\left(\sqrt{\text{cos}{\theta\:}_{i}}-\sqrt{\text{cos}{\theta\:}_{r}}{e}^{j{\Phi\:}\left(x\right)}\right)}$$

where;

7 $$\:{\Phi\:}\left(x\right)=\:{k}_{0}\:\left(sin\:{\theta\:}_{i}-\:sin\:{\theta\:}_{r}\right)\:x$$

**Fig. 2:**
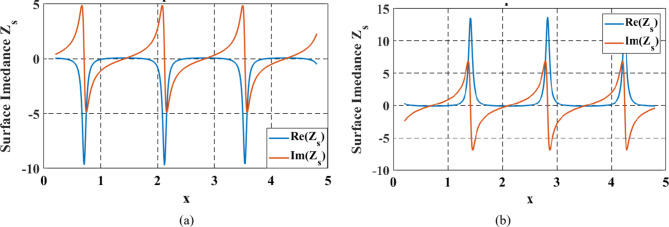
Impedance of perfect reflector with $\theta_{i}=0^\circ\:$ and $\theta_{r}=45^\circ\:$ for (**a**) TM and (**b**) TE polarization.

The required impedance modulation of an ideal metasurface that would reflect a normally incident wave to a reflection angle of $\:{\theta\:}_{r}\:=\:45^\circ\:$, for TM and TE polarizations are shown in Fig. 2a, b, respectively. From these figures, it can be clearly shown that the real component of the modulated impedance should exhibit both positive and negative values across different regions of the surface. This implies that incorporating alternating lossy and active regions is essential to maintain the desired locality property.

Now, consider a grounded dielectric substrate, this substrate has a thickness of $\:d$ and a relative permittivity $\:{\epsilon\:}_{r}$. On top of this substrate, a lossless surface impedance $\:j\:Xs$ changes periodically in the $\:x-$direction, with period $\:L$. The surface admittance $\:jBs\:=\:j\left(-\frac{1}{Xs}\right)$ is represented by a series expansion $\:P$ of Fourier coefficients as:

8 $$\:j{B}_{s}=j{B}_{0}\left[1+\sum\:_{p=1}^{p}{M}_{p}\:\text{c}\text{o}\text{s}\left(2p\pi\:\frac{x}{L}\right)+\:{N}_{p}\:\text{s}\text{i}\text{n}\left(2p\pi\:\frac{x}{L}\right)\right]$$

where, $\:j{B}_{0}$ denotes the average admittance, and $\:{M}_{p}$ and $\:{N}_{p}$ are constant real-valued coefficients. Therefore, the boundary condition on z = 0 is:

9 $$\:\widehat{z}\:\times\:\:\left({H}_{t}\left|z={o}^{+}\right.-\:{H}_{t}\left|z={o}^{-}\right.\right)=\:\frac{j{B}_{s}}{{\eta\:}_{o}}\:{E}_{t}\left|z=0\right.$$

The analysis method is based on eigenvalue technique for simple, sinusoidally modulated surfaces. However, it has been adapted to modify the case of metasurfaces with arbitrary periodic profiles illuminated by incident plane waves. The metasurface boundary condition is represented using a series expansion for TE and TM Polarizations.

Both polarizations have a similar way of expressing the boundary conditions for the metasurface. Based on the Floquet’s theorem, the tangential component of the total electric field and total magnetic field, for the TM polarization as an example, can be expressed as series expansion of $\:{n}^{th}$ order Floquet modes^20^. Then, The boundary condition in (9) is applied to give a general series expansion in terms of the unknown Floquet coefficients as the following:

10 $$\:\sum\:_{n=\:-{\infty\:}}^{{\infty\:}}{A}_{\:\text{x},\:n}\:{E}_{x,n}\:{e}^{-j{k}_{0}{\beta\:}_{n,}x}=\:{B}_{s}\sum\:_{n=\:-{\infty\:}}^{{\infty\:}}{E}_{\:\text{x},n}\:{e}^{-j{k}_{0}{\beta\:}_{n}x}$$

where $\:{B}_{s}$ is given by (8) and the coefficients of the $\:{n}^{th}$-order Floquet mode $\:{A}_{x,n}$ and$\:\:{\xi\:}_{n}$ are given as:

11a $$\:{A}_{x,n}=\:-\frac{1}{{\xi\:}_{n}}+\:\frac{{\epsilon\:}_{r}\:cot\left({k}_{0}\sqrt{{\epsilon\:}_{r}-{\beta\:}_{n}^{2}\:}\:d\right)}{\sqrt{{\epsilon\:}_{r}-{\beta\:}_{n}^{2}\:}\:}$$

11b $$\:{\xi\:}_{n}=\:\left\{\:\:\begin{array}{c}j\text{cos}{\theta\:}_{i}\frac{{\Gamma\:}+1}{{\Gamma\:}-1}\:\:\:\:\:\:\:\:\:\:n=0\:\:\:\:\\\:{a}_{n}\:\:\:\:\:\:\:\:\:\:\:\:\:\:\:\:\:\:\:\:\:\:\:\:\:\:\:\:\:\:n\ne\:0\:\:\:\:\:\:\:\end{array}\right.$$

where $\:{\Gamma\:}$ is the specular reflection coefficient for $\:{\theta\:}_{i}=0^\circ\:$. $\:{a}_{n}=\sqrt{{{\beta\:}_{n}}^{2}-1}.$ It should be noted that when $\:\left({\beta\:}_{n}>1\:\right)$ the field of the $\:{n}^{th}$-order Floquet mode is evancent mode which contribute to surface wave and the field propagates to the desired reflection angle only if $\:{\beta\:}_{n}<1$

12 $$\:{\beta\:}_{n}=\:{\beta\:}_{0}+\:\frac{n\pi\:}{L}$$

where $\:{\beta\:}_{n}$ is the propagation constant of the $\:{n}^{th}$ mode, normalized to $\:{k}_{0}$ and $\:{\beta\:}_{0}=\text{sin}{\theta\:}_{i}$.

By following the same procedures from (8) to (12) a similar series expansion as in (10) can be rewritten for the TE polarization case. To theoretically solve Eq. (10), an infinite system of equations can be expressed as:

13 $$\:{Q}_{P\:}{E}_{n-P\:}+\:{Q}_{P-1\:}{E}_{n-P+1\:}+\:\cdots\:\:+\:\:{D}_{n\:}{E}_{n\:}+\cdots\:\:{+{Q}_{P-1}^{{\prime\:}}E}_{n+P-1}+\:{{Q}_{P}^{{\prime\:}}E}_{n+P}=0$$

where *Q*_*p*_ = *M*_p_ + j*N*_p_, *M*_p_ and *N*_p_ are the modulation parameters, Q'_*p*_ is the complex conjugate of *Q*_p_ and $\:{D}_{n}$ is given as:

14 $$\:{D}_{n}=2\left(1-\frac{{A}_{n,x}}{{B}_{0}}\right)$$

By setting $\:{E}_{n\:}=0$ for $\:\left|n\right|\:>\:\text{K}$ and taking into account a high number of Floquet modes K to provide a valid approximation, we can truncate the infinite system in (13) and creating a system of $\:2\text{K}\:+\:1$ equations that approximates the metasurface’s electromagnetic response in a discrete form as:

15 $$\:\mathbf{D}\left[\begin{array}{c}{E}{-k}\\\:\vdots\\\:{E}{-1}\\\:{E}{0}\\\:{E}{1}\\\:\vdots\\\:{E}_{k}\end{array}\right]=\left[\begin{array}{c}0\\\:\vdots\\\:0\\\:0\\\:0\\\:\vdots\\\:0\end{array}\right]$$

In normalized form, (15) can be written as:

16 $$\:\stackrel{\sim}{\text{D}}\:\left[\begin{array}{c}{e}_{-k}\\\:\vdots\\\:{e}_{-1}\\\:{D}_{0}\\\:{e}_{1}\\\:\vdots\\\:{e}_{k}\end{array}\right]=\:\:\:\:\left[\begin{array}{c}0\\\:\vdots\\\:0\\\:{Q}_{P}^{{\prime\:}}\\\:\vdots\\\:{Q}_{1}^{{\prime\:}}\\\:0\\\:{Q}_{1}\\\:\vdots\\\:{Q}_{P}\\\:0\\\:\vdots\\\:0\end{array}\right]$$

where $\:en\:=\frac{{E}_{n}}{{E}_{0}}$. The unknown variables of the system are $\:{D}_{0}\:=\:{D}_{0}\left(\varGamma\:\right)$ and the field coefficients $\:{e}_{-k}$,. . ., $\:{e}_{-1}$, e_1_,. . ., $\:{e}_{k}$.

where,

17 $$\:\stackrel{\sim}{D}=\:\left[\begin{array}{cc}{D}_{11}&\:{D}_{12}\\\:{D}_{21}&\:{D}_{22}\end{array}\right]$$

where $\:{D}_{11}$ and $\:{D}_{22}$ are $\:K\:x\:K$ diagonal matrices populated with elements of the sequence $\:Q$ with specific indexing. Also, $\:{D}_{12}$ and $\:{D}_{21}$ are $\:K\:x\:K$ matrices filled with zeros initially, then populated with elements of $\:Q$ using the defined indexing within the loops. This system of equations in (16) can be solved numerically to determine the unknown modulation coefficients *M*_p_ and *N*_p_.

### Genatic alogrithm in metasurface design

**Fig. 3:**
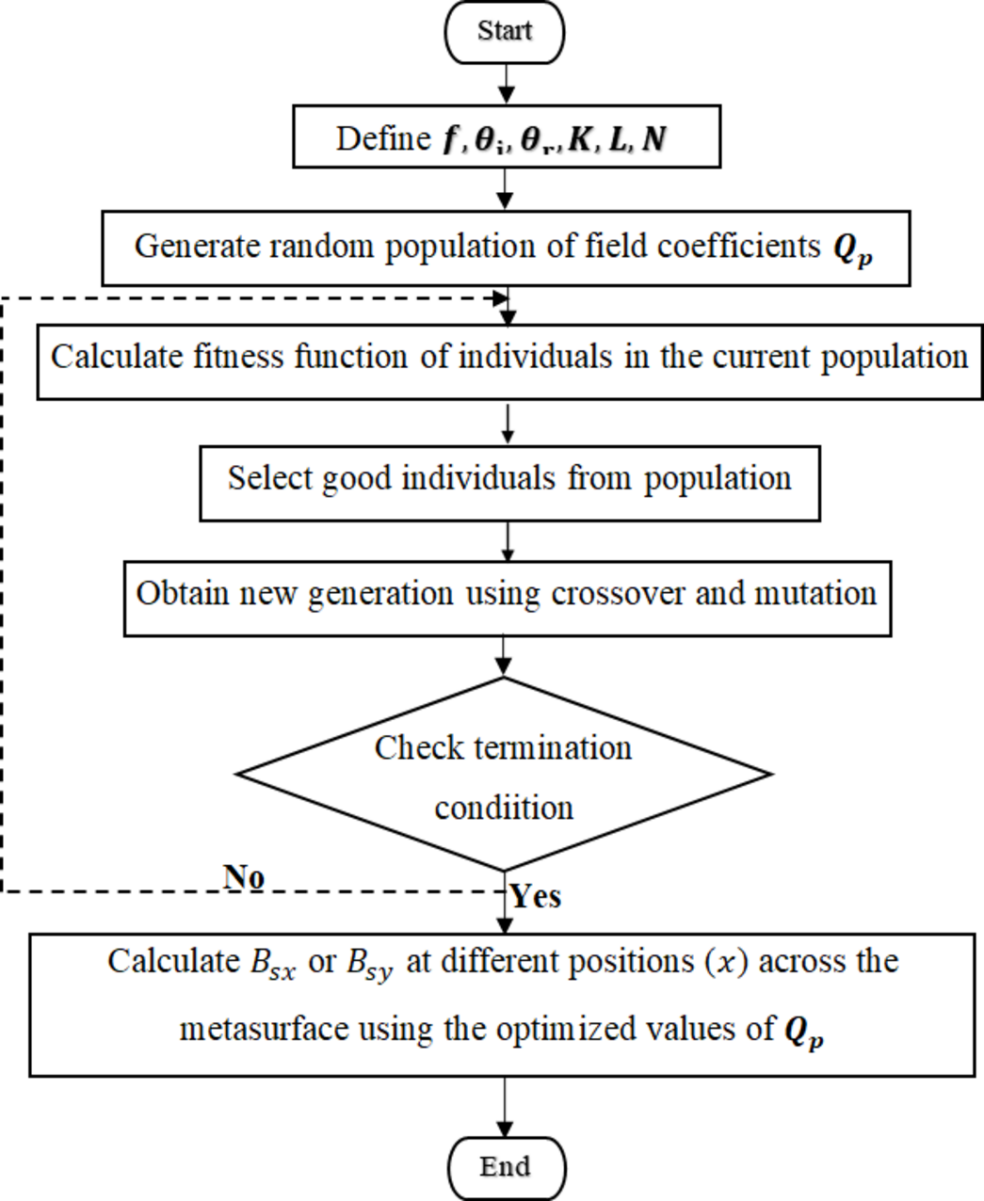
Flowchart for the design procedures of anomalous reflector metasurface using GA.

In order to achieve the desired surface admittance $\:{jB}_{sx}$ and $\:{jB}_{sy}$for the proposed anomalously reflecting metasurface, it is necessary to determine the modulation parameters $\:Q$. These modulation parameters are obtained through the use of a Genetic Algorithm (GA). The GA optimization process begins with an initial population consisting of several candidate solutions, referred to as chromosomes^23,24^. These parent chromosomes undergo various operations such as combination, crossover, or mutation, resulting in a new set of chromosomes for the next generation. As the arrangement progresses, the chromosomes are evaluated based on how well they improve the fitness between the obtained radiation pattern and the required mask. The higher-ranking chromosomes are selected to proceed to the next generation. Once the new generation is formed, the fitness of its chromosomes is evaluated, and the process continues until a convergence condition is met. The algorithm terminates when the fitness value for the best point in the current population is equal to or falls below the fitness limit. The key steps of the genetic algorithm are depicted in Fig. 3.

18 $$\:fitness=\left|{D}_{o}-E\left(K+1\right)\right|+\left|E\left(K\right)\right|$$

**Table 1:** Optimized modulation parameters of the surface admittance required to reflect wave to $\:45^\circ.$

Modulation parameter	*B*_sx_	*B*_sy_
*B*_o_	3.609	4.929
*Q*_1_	0.253 + *j* 0.532	−0.302 + *j* 0.401
*Q*_2_	−0.125 − *j* 0.300	0.145 − *j* 0.311
*Q*_3_	0.180 + *j* 0.139	0.008 − *j* 0.015
*Q*_4_	−0.134 + *j* 0.077	0.115 − *j* 0.082
*Q*_5_	−0.089 + *j* 0.034	0.102 + *j* 0.072

By applying the overall analysis, an anomalous reflector that reflects a normal incident wave ($$\:{\theta\:}_{i}$$$$\:=\:0^\circ\:$$) with to a direction of $$\:{\theta\:}_{r}=45^\circ\:$$ at frequency of 8 GHz can be designed. To obtain the appropriate values for parameters $$\:{B}_{0}$$, P, $$\:{Q}_{1}$$, …, $$\:{Q}_{P}$$, the Eq. (16) is numerically solved at each step of the parametric analysis. The goal is to find values that satisfy $$\:{E}_{-1}$$$$\:=\:0$$. The resulting values are presented in Table 1, where $$\:{jB}_{sx}\left(x\right)$$ and $$\:j{B}_{sy}\left(x\right)$$ represent the admittances for TM and TE polarizations, respectively.

### Elliptical unit cell design

**Fig. 4:**
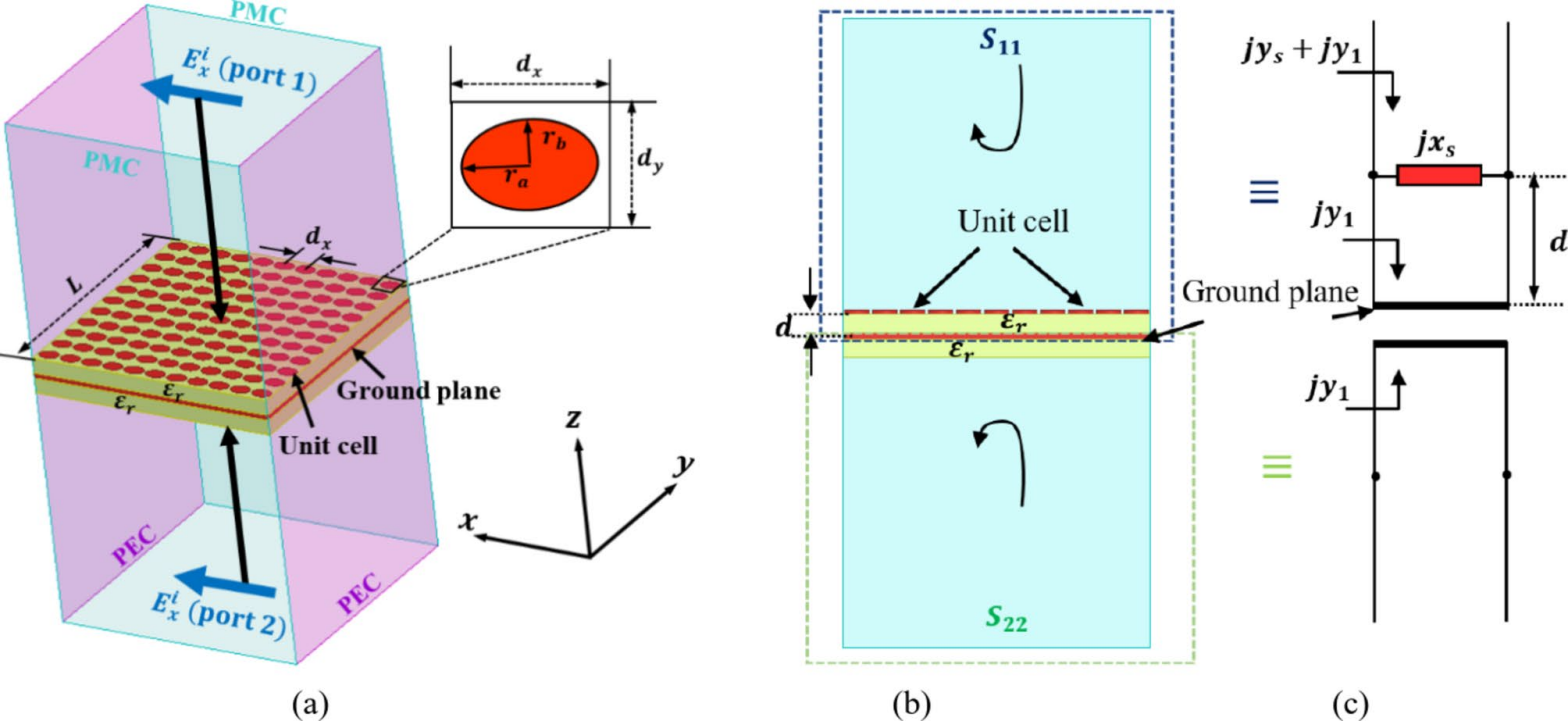
Representation of the metasurface array. (**a**) Unit cell inside periodic boundary. (**b**) Side view. (**c**) Equivalent transmission line model.

The previous section showed how to obtain the surface admittance $\:{jB}_{sx}$ and $\:{jB}_{sy}$ which is required to reflect the normal incident wave to a direction $\:{\theta\:}_{r}=45^\circ\:$. The following step is to demonstrate a method for designing the required reflecting surface with a prescribed surface admittance profile. To achieve this, an impedance surface of upper and lower structures of dimension $\:L\times\:L$ is introduced as shown in Fig. 4a, b. The upper structure is composed of an array of elliptical patches of $\:N\times\:N$ configuration, with a spacing of *dx* = L/N between adjacent patches. The elliptical patches are printed on a dielectric substrate of thickness $\:d$ and permittivity$\:{\epsilon\:}_{r}$. The backside of the substrate is backed with a ground plane which is also placed on a dielectric substrate with the same thickness and permittivity to form the lower structure. The lower configuration is placed to aid in the calculation of the surface admittance of the patches. This structure can be modeled using a transmission line model^25^. The impedance sheet of the elliptical patches acts as a shunt inductor ($\:j{x}_{s}$) placed at a distance $\:d$ from a short-circuited dielectric loaded transmission line with admittance$\:\left(j{y}_{1}\right)$. The total admittance of the upper structure is given by $\:\left(j{y}_{s}+j{y}_{1}\right)$ as shown in Fig. 4c. On the other hand for the lower structure, the ground plane backed by dielectric is modeled as a shorted transmission line with its length equal to the substrate thickness with admittance $\:\left(j{y}_{1}\right)$. The normalized surface admittance for the upper and lower structures are given as:

19 $$\:{Y}_{upper}=j{y}_{s}+j{y}_{1}$$

20 $$\:{Y}_{lower}=j{y}_{1}$$

subtracting (19) from (20) to get the surface admittance of the patches:

21 $$\:j{y}_{s}={Y}_{upper}-{Y}_{lower}$$

To account the interaction between this structure and a normally incident plane wave, the structure is bounded by the PMC-PEC boundaries and illuminated by Floquet ports with normal incident wave at both ends for both TM- and TE-polarizations. By adjusting of the ellipse dimensions $\:{r}_{a}$ and $\:{r}_{b}$, the reflection coefficients $\:{S}_{11}$ and $\:{S}_{22}$ of the two incident waves can be controlled. Then, the surface admittance $\:{jB}_{s}$ can be easily extracted from the reflection coefficients as:

22 $$\:{{jB}_{s}=Y}_{upper}-{Y}_{lower}=\frac{1-{S}_{11}}{1+{S}_{11}}-\frac{1-{S}_{22}}{1+{S}_{22}}$$

**Fig. 5:**
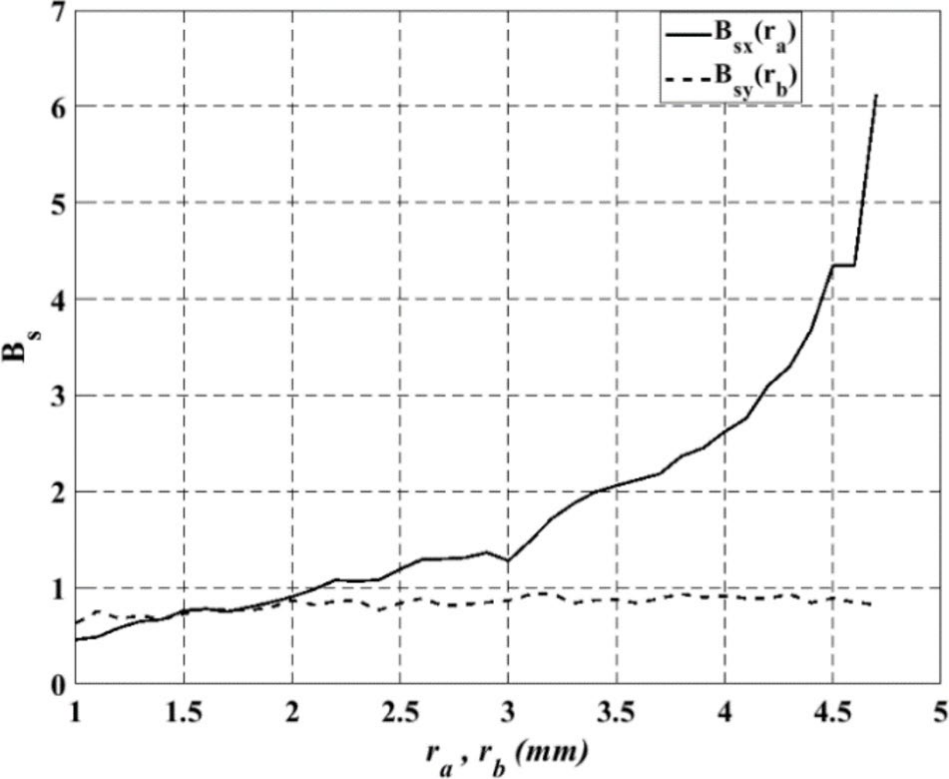
Surface admittance of the reflected signal from the reflecting surface in periodic boundaries.

To implement the anomalous reflector, the periodic modulation is discretized into eleven segments ($\:N=11$) and approximate it using a collection of elliptical patches with different dimensions. Figure 5 shows the surface admittance of the reflecting surface as a function of $\:{r}_{a}$ and $\:{r}_{b}$ at constant values of $\:{r}_{b}$ and $\:{r}_{a}$, respectively. To perform an anomalous reflector design which is required to reflect a normal incident wave $\:\left({\theta\:}_{i}=0^\circ\:\right)$ to a direction $\:{\theta\:}_{r}=45^\circ\:$ at frequency $\:f$ = 10 GHz, the modulation period according to the generalized reflection law ($\:L=\lambda/{\left|\text{sin}{\theta\:}{r}-\text{sin}{\theta\:}{i}\right|}$), $\:L=53\:\text{m}\text{m}$. The reflecting elements are printed on a grounded Rogers3010 substrate with a dielectric constant $\:{\epsilon\:}_{r}=10.2$ and a dielectric thickness $\:d$=1.27 mm. The periodicity in $\:x-y$ plane is $\:dx=dy\:=\:4.82\:\text{m}\text{m}$. Hence, the maximum allowable values for the lengths of the reflecting element are chosen to be $\:4.75$ mm. Thus, $\:{r}_{a}$ and $\:{r}_{b}$ are changed between 1 mm and 4.7 mm to generate a lookup table for the surface admittance of the TM or TE polarized waves. Then, the obtained modulation parameters given by GA algorithm is used to calculate the surface admittance values$\:{\:\stackrel{\prime }{B}}_{sx}$ and $\:{\:\stackrel{\prime }{B}}_{sy}$. By calculating the relative error between the analytical and numerical values of surface admittance in both x and y directions, the dimensions of the elliptical patch can be obtained corresponding to the minimum relative error. The relative error between analytical ($\:{\:\stackrel{\prime }{B}}_{sx}$,$\:\:{\:\stackrel{\prime }{B}}_{sy}$) and numerical ($\:{B}_{sx},{B}_{sy}$) values is given as^23^:

23 $$\:error=\frac{\left|{B}_{sx}-{\:\stackrel{\prime }{B}}_{sx}\right|}{{B}_{sx}}+\frac{\left|{B}_{sy}-{\:\stackrel{\prime }{B}}_{sy}\right|}{{B}_{sy}}$$

as a function of $\:{r}_{a}$ and $\:{r}_{b}$.

### Numerical simulation

**Fig. 6:**
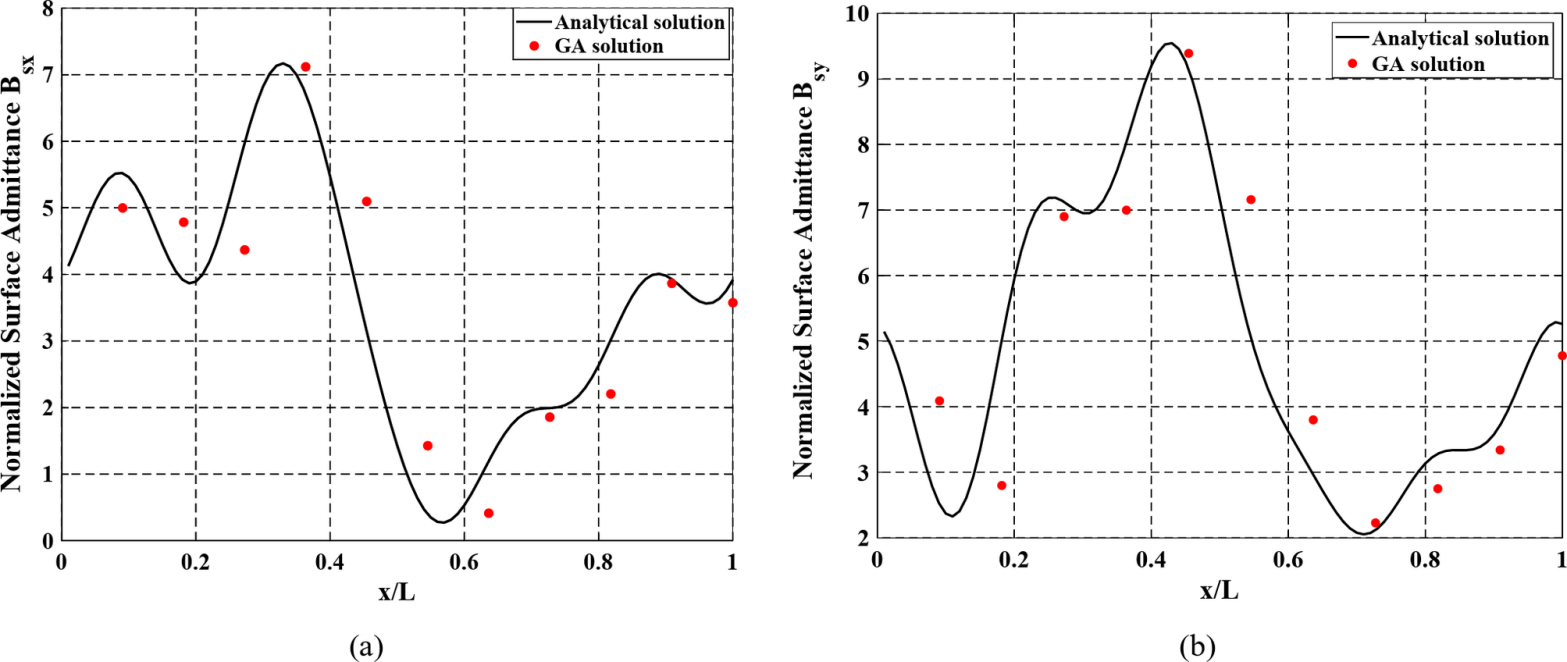
Normalized surface admittance across the modulation period. (**a**) TM polarization. (**b**) TE polarization.

By applying the analytical solution, the normalized surface admittance across the modulation period is shown in Fig. 6. The dimensions of the elliptical patches constituting the metasurface pattern, extracted by the periodic boundary analysis are shown in Table 2.

**Table 2:** Dimensions of the elliptical patches of the reflecting metasurface according to $\:{B}_{sx}$ and $\:{B}_{sy}$

Patch number	r_a_(mm)	r_b_(mm)	B_X_	B_Y_
1	2.20	2.25	4.9981	4.0888
2	2.00	2.15	4.7838	2.7979
3	2.25	2.15	4.3673	6.8996
4	2.20	2.25	7.1174	6.9974
5	2.25	2.35	5.0955	9.3894
6	2.35	2.00	1.4272	7.1580
7	2.30	1.65	0.4127	3.7983
8	1.10	1.30	1.8566	2.2281
9	2.05	1.65	2.2043	2.7488
10	1.90	2.05	3.8654	3.3392
11	2.10	2.15	3.5751	4.7779

**Fig. 7:**
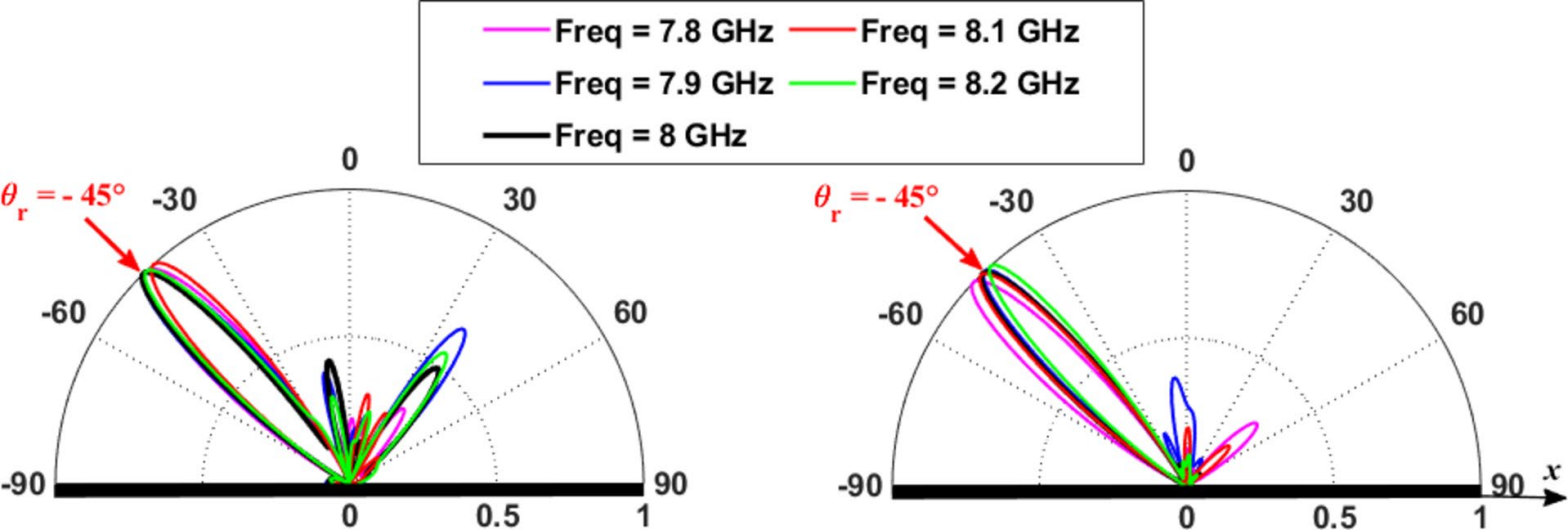
Normalized 2D radiation pattern for an anomalous metasurface of reflective elliptical patch for (**a**) TM polarization. (**b**) TE polarization at the frequency band of 7.8$\:-$8.2 GHz.

**Fig. 8:**
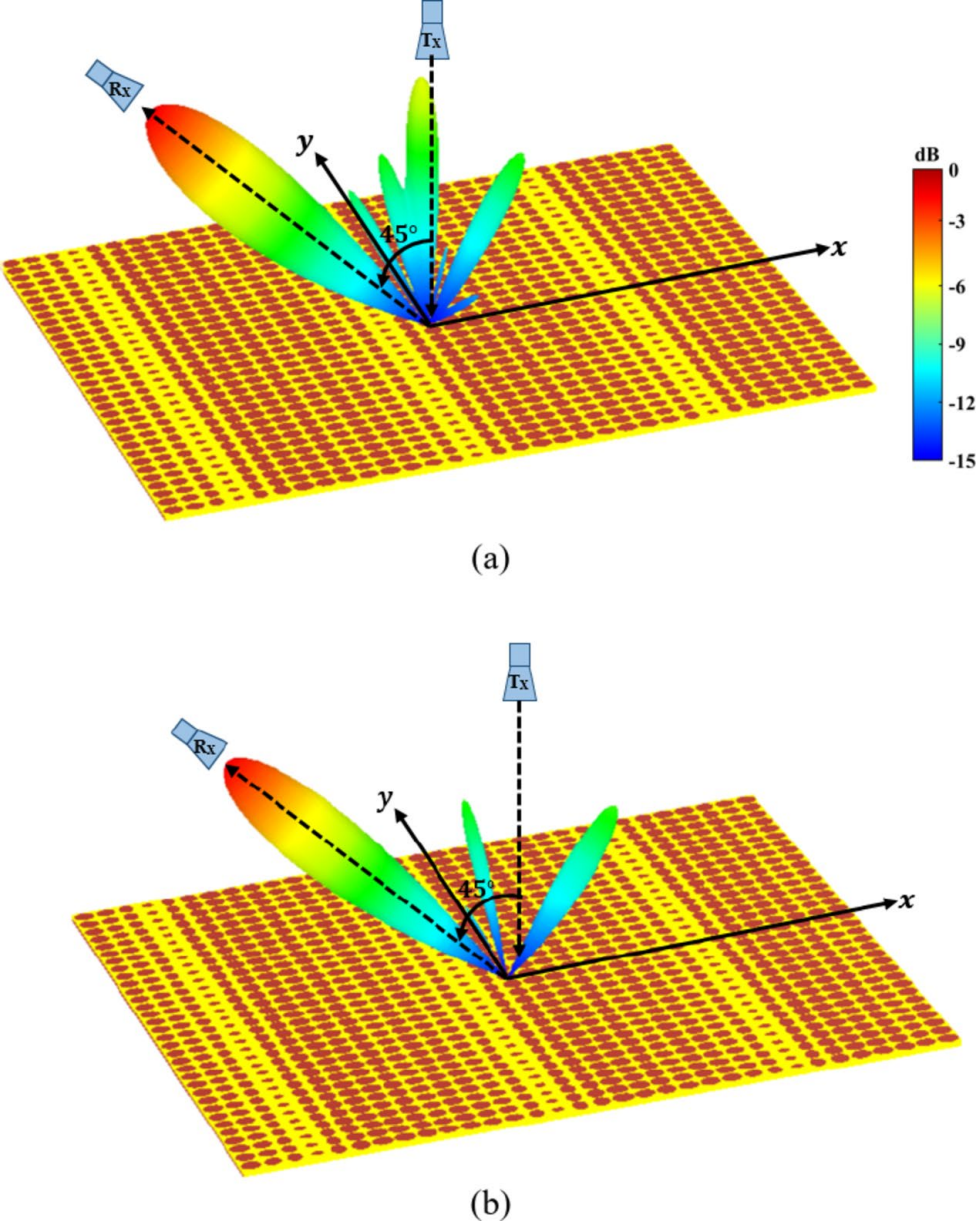
Normalized 3D radiation pattern for an anomalous metasurface of reflective elliptical patch at 8 GHz, (**a**) TM polarization. (**b**) TE polarization.

The simulated 2D radiation patterns for the designed reflector for TM and TE polarizations for frequency band of 7.8 GHz to 8.2 GHz are shown in Fig. 7. For the TM polarization case it can be noted that the reflections in the specular directions are higher compared to the TE polarization. Also, 3D normalized radiation pattern at 8 GHz is shown in Fig. 8. The presented radiation pattern shows a main reflected lobe are almost at $\:45^\circ\:$.

## Experimental Verification

**Fig. 9:**
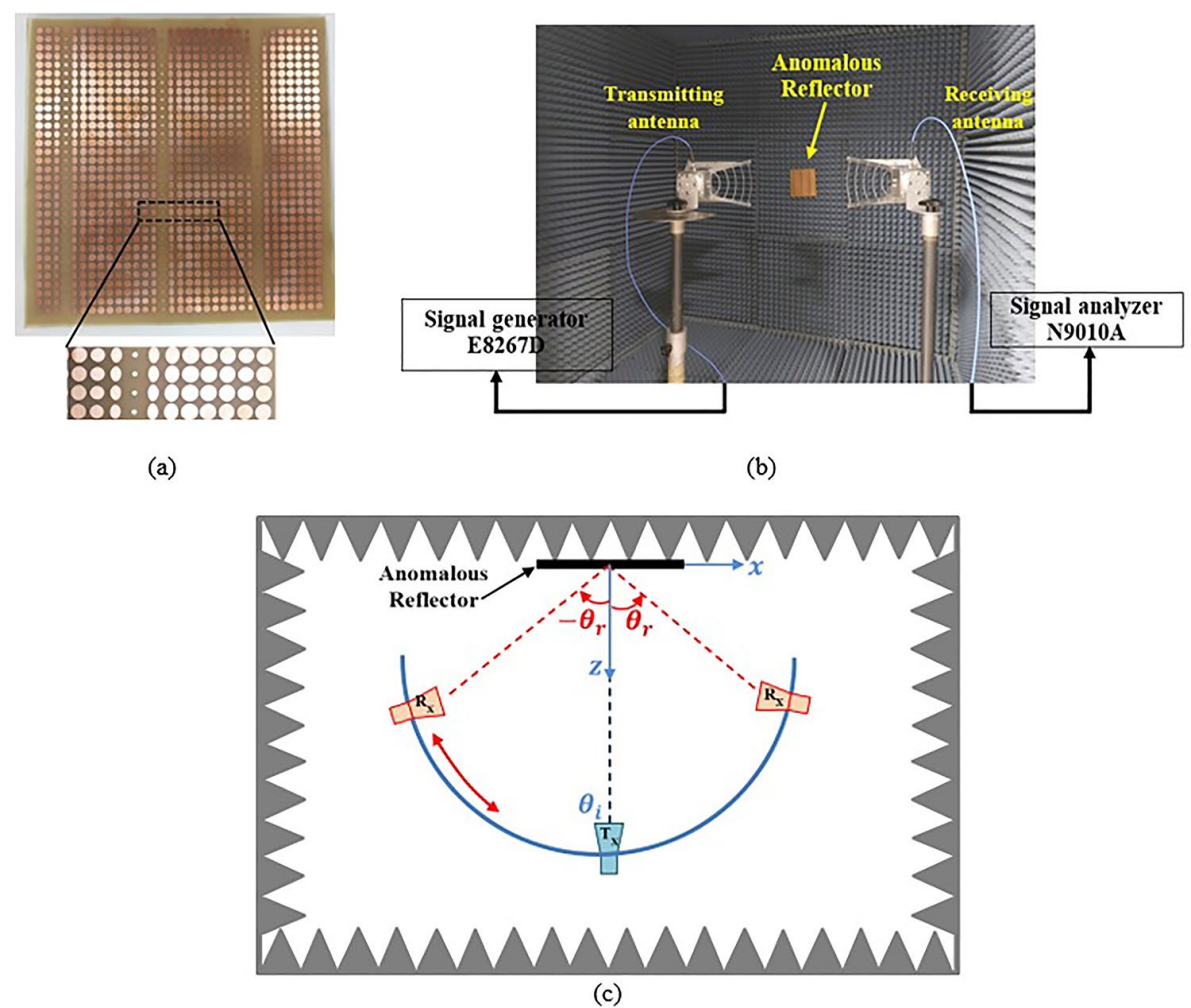
(**a**) Fabricated prototype of the proposed anomalous reflector. (**b**) Experimental setup in the anechoic chamber. (**c**)Top view of the experimental setup.

**Fig. 10:**
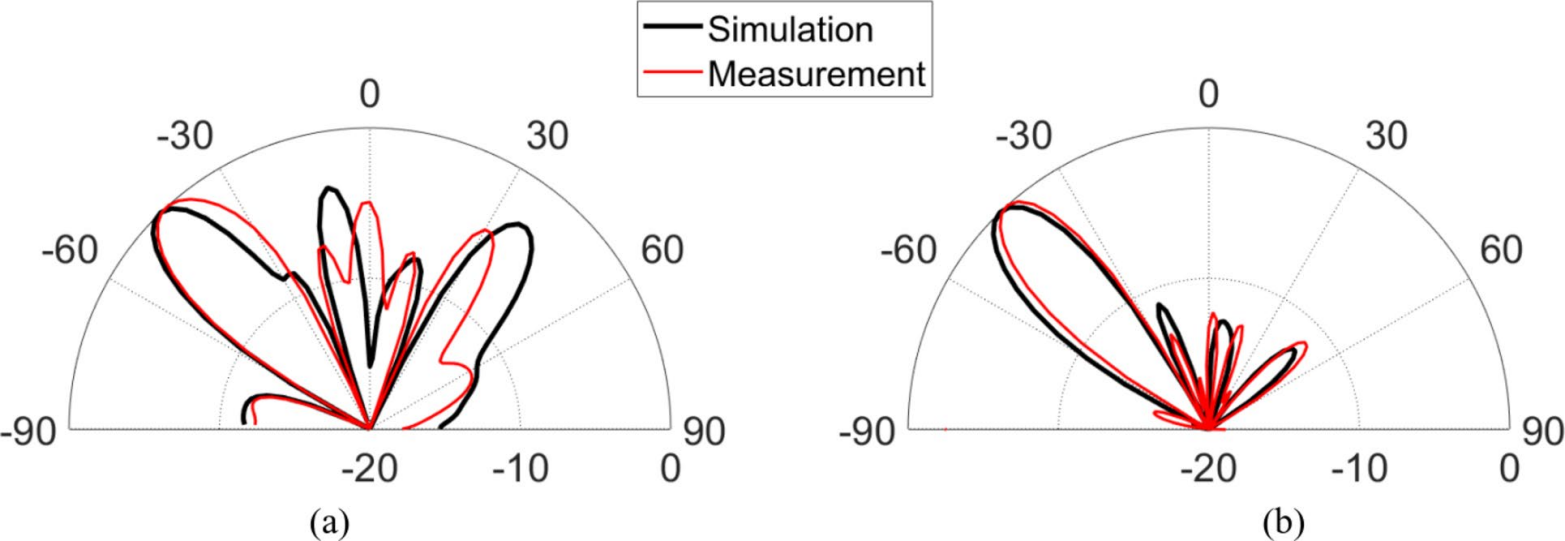
Measured and simulated radiation pattern of the reflected power of the proposed anomalous reflector for (**a**) TM and (**b**) TE polarization at 8 GHz.

**Fig. 11:**
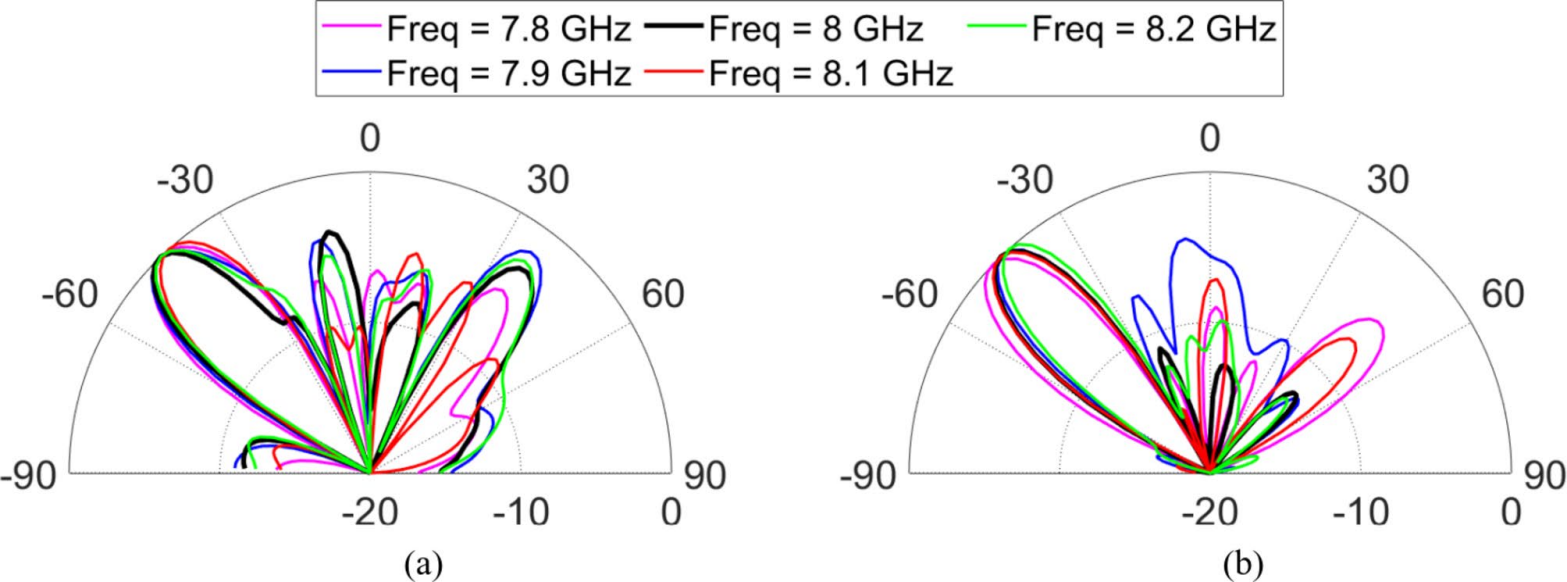
Measured radiation pattern of the reflected power of the proposed anomalous reflector at different frequencies from 7.8–8.2 GHz for (**a**) TM polarization and (**b**) TE polarization.

Figure 9 shows the experimental setup used to evaluate the reflection characteristic of anomalous reflectors. For verification of the simulated CST results, the proposed anomalous reflector of (159 × 159) mm^2^ is fabricated as shown in Fig. 9 (a). An in-house EM-shielded anechoic chamber with dimensions (3.5 × 4 × 5) m^3^ is used for testing the manufactured reflector. An experimental setup is established to prove the reflection ability of the manufactured sheet as shown in Fig. 9 (b). Two identical standard gain horn antennas (A-INFO LB-60180-10) offering 12 dBi gain at 8 GHz are placed in front of the fabricated reflector sheet. One of the two horn antenna, work as a transmitter and is connected to Agilent E8267D signal generator and the other one, as a receiver and is connected to Agilent EXA signal analyzer N9010A for measuring the amplitude of the reflected wave for all available observation angles $\:{\theta\:}_{r}$. The distance between the reflector and the transmitting antenna was equal to 100 cm. The measurements are carried out at 8 GHz with the receiving antenna moving at 2° resolution across azimuth ∈ [− 90°, 90°] as shown in Fig. 9c. The normalized scattering patterns acquired from the measurements are compared with the CST simulated outcomes, as illustrated in Fig. 10. The obtained measurement data reveals good agreement between the simulation and measurement results at the desired reflection angle. Also, in the desired maximum measured power, a slight deviation by a 3° can be observed between the simulation and measurement results. Additionally, we present the measured results of the normalized scattering patterns for the frequency range of 7.8–8.2 GHz in Fig. 11 to evaluate the frequency dependency of the designed metasurface for both TM and TE polarizations. From Fig. 11, it can be noted that the designed anomalous reflector is narrowband within a 400 MHz range for the TE and TM cases.

## Conclusion

This paper describes an efficient approach for synthesizing metasurfaces that exhibit anomalous reflection. The approach involves rapidly calculating the electromagnetic response of periodically modulated surface impedance, considering accurate analytical calculations of Floquet modes. By expressing the metasurface’s boundary condition as a solvable matrix equation with a small number of unknown variables, the method significantly outperforms full-wave computational methods in terms of speed. Using the analytical technique, it becomes straightforward to derive appropriate periodic modulation surface impedance using genetic algorithm (GA) optimization technique. To examine the proposed analytical technique, an anomalous reflector is designed to reflect normal incident waves to an angle of 45˚ for both TM and TE polarization cases at operating frequency of 8 GHz for communication systems. The discrete implementation of the reflector was achieved through an array of elliptical patches. For validation of the simulated results, a reflector sheet is fabricated and measured at the frequency band from 7.8 GHz to 8.2 GHz. Good agreement between the simulated and the measured results is obtained in the desired reflection angles.

## Data Availability

The datasets used and/or analysed during the current study available from the corresponding author on reasonable request.

## References

[1] Liu, S. et al. A review of anomalous refractive and reflective metasurfaces. *Phys. Rev. B*. **94**, 1–12 (2016).

[2] Wong, A. M. H. & Eleftheriades, G. V. Perfect anomalous reflection with a bipartite Huygens’ metasurface. *Phys. Rev. X*, **8**, 1–8 (2018).

[3] Rabinovich, O. & Epstein, A. Analytical design of printed circuit board (PCB) metagratings for perfect anomalous reflection. *IEEE Trans. Antennas Propag.* **66** (8), 4086–4095 (2018).

[4] Modi, A., Balanis, C., Birtcher, C. & Shaman, H. New class of RCS-reduction metasurfaces based on scattering cancellation using array theory. *IEEE Trans. Antennas Propag.* **67** (1), 298–308 (2019).

[5] Rahmanzadeh, M. & Khavasi, A. Perfect anomalous reflection using a compound metallic metagrating. *Opt. Exp.* **28** (11), 16439 (2020). 10.1364/OE.393137 32549467

[6] Islam, N. & Choi, S. Compact folded dipole metasurface for high anomalous reflection angles with low harmonic levels. *Sci. Rep.* **10**, 1–13 (2020). 10.1038/s41598-020-75230-2 PMC7582140 33093589

[7] Diaz-Rubio, A. & Tretyakov, S. Macroscopic modeling of anomalously reflecting metasurfaces: Angular response and far-field scattering. *IEEE Trans. Antennas Propag.* **69** (10), 6560–6571 (2021).

[8] Liu, Y. & Sarris, C. D. Efficient propagation modeling for communication channels with reconfigurable intelligent surfaces. *IEEE Antennas Wirel. Propag. Lett.* **21** (10), 2120–2124 (2022).

[9] Vuyyuru, S., Valkonen, R., Kwon, D. & Tretyakov, S. Efficient Anomalous Reflector Design Using Array Antenna Scattering Synthesis. *IEEE Ant Wire Prop. Lett.* **22** (7), 1711–1715 (2023).

[10] Liu, X. et al. Perfect anomalous reflection and refraction utilizing binary Pancharatnam–Berry phase elements based metasurfaces. *J. Phys. D Appl. Phys.* **53** (6), 1–8 (2020).

[11] Islam, N. A. & Choi, S. Compact folded dipole metasurface for high anomalous reflection angles with low harmonic levels. *Sci. Rep.* **10** (1), 1–13 (2020). 10.1038/s41598-020-75230-2 PMC7582140 33093589

[12] Tsilipakos, F. et al. Intelligent metasurfaces with continuously tunable local surface impedance for multiple reconfigurable functions. *Phys. Rev. Appl.* **11** (4), 1–10 (2019).

[13] Sun, Y. et al. Broadband anomalous reflective metasurface for complementary conversion of arbitrary incident polarization angles. *Opt. Exp.* **29** (23), 1–11 (2021). 10.1364/OE.444128 34808894

[14] Díaz-Rubio, A., Asadchy, V., Elsakka, A. & Tretyakov, S. From the generalized reflection law to the realization of perfect anomalous reflectors. *Sci. Adv.* **3** (8), 1–10 (2017). 10.1126/sciadv.1602714 PMC5553823 28819642

[15] Yepes, C., Faenzi, M., Maci, S. & Martini, E. Perfect non-specular reflection with polarization control by using a locally passive metasurface sheet on a grounded dielectric slab. *Appl. Phys. Lett.* **118** (23), 1–8 (2021).

[16] Shi, H. et al. Gradient Metasurface with Both Polarization Controlled Directional Surface Wave Coupling and Anomalous Reflection. *IEEE Ant Wire Prop. Lett.* **14**, 104–107 (2015).

[17] Barnard, W., Odendaal, J. & Joubert, J. Anomalous Reflection From a Phase Gradient Metasurface With Arbitrary Incident Angle. *IEEE Access.* **11**, 18385–19390 (2023).

[18] Malleboina, R., Dash, J. & Sarkar, D. Design of Anomalous Reflectors by Phase Gradient Unit Cell-Based Digitally Coded Metasurface. *IEEE Ant Wire Prop. Lett.* **22** (9), 2305–2309 (2023).

[19] Movahediqomi, M., Ptitcyn, G. & Tretyakov, S. Comparison Between Different Designs and Realizations of Anomalous Reflectors for Extreme Deflections. *IEEE Trans. Ant Prop.* **71** (10), 1–9 (2023).

[20] Raptis, S. & Yioultsis, T. Synthesis of Polarization-Independent Perfect Anomalous Reflectors via Modulated Metasurfaces and an Analytical Design Model, IEEE Trans. On Microwave Theory and Techniques, Vol. 71, No. 11, Nov. (2023).

[21] Asadchy, V. S. et al. Perfect control of reflection and refraction using spatially dispersive metasurfaces. *Phys. Rev. B Condens. Matter*. **94** (7), 1–14 (2016).

[22] Kwon, D. Lossless Scalar Metasurfaces for Anomalous Reflection Based on Efficient Surface Field Optimization. *IEEE Ant Wire Prog Lett.* **17** (7), 1149–1152 (2018).

[23] Borgese, M., Costa, F., Genovesi, S., Monorchio, A. & Manara, G. Optimal Design of Miniaturized Reflecting Metasurfaces for Ultra-Wideband and Angularly Stable Polarization Conversion. *Sci. Rep.* **8**, 1–11 (2018). 10.1038/s41598-018-25934-3 PMC5955921 29769556

[24] Abdullah, T., Imran, H., Chowdhury, S. & Haque, M. A Genetic Algorithm Approach for A Broadband Reflector Design, IEEE Annual Computing and Communication Workshop and Conference (CCWC), pp. 597–602, (2024).

[25] Kosulnikov, S., Wang, X. & Tretyakov, S. Discrete-Impedance Metasurfaces for Wireless Communications in D-Band. *IEEE Trans. Ant Prop.* **72** (1), 1–8 (2024).

